# Synovial Fluid Metabolome Can Differentiate between Healthy Joints and Joints Affected by Osteoarthritis in Horses

**DOI:** 10.3390/metabo13080913

**Published:** 2023-08-04

**Authors:** Fulvio Laus, Rodolfo Gialletti, Marilena Bazzano, Luca Laghi, Fabrizio Dini, Andrea Marchegiani

**Affiliations:** 1 School of Biosciences and Veterinary Medicine, University of Camerino, 62024 Macerata, Italy; fulvio.laus@unicam.it (F.L.); fabrizio.dini@unicam.it (F.D.); andrea.marchegiani@unicam.it (A.M.); 2 Department of Veterinary Medicine, University of Perugia, 06123 Perugia, Italy; rodolfo.gialletti@unipg.it; 3 Centre of Foodomics, Department of Agro-Food Science and Technology, University of Bologna, 40100 Bologna, Italy; l.laghi@unibo.it

**Keywords:** osteoarthritis, metabolome, H-NMR, horse

## Abstract

Osteoarthritis (OA) is a common cause of lameness in sport horses with a significant economic impact. The prevention of OA is crucial since no effective treatment is available. This study aimed to apply untargeted metabolomic analysis to investigate the differences in synovial fluid (SF) composition between healthy and OA-affected joints in horses. SF collected from healthy (n.8) and OA (n.11) horses was analyzed using H-NMR analysis. Metabolomic analysis allowed 55 different metabolites to be identified and quantified in SF samples. Nineteen metabolites were found to be differently concentrated in OA compared to control horses. Synovial fluids from the OC group were found to be higher in 1,3-dihydroxyacetone but lower in tryptophan, phenylalanine, tyrosine, uridine, creatinine, creatine, glycine, choline, asparagine, glutamine, arginine, 3-hydroxybutyrate, valine, 2-hydroxyisovalerate, α-ketoisovaleric acid, 3-methyl-2-oxovalerate, 3-hydroxyisobutyrate, isoleucine, and methionine compared to the controls. A variety of SF metabolites significantly changed following joint disease, demonstrating the complex mechanism underlying osteoarthritis in horses and highlighting the value of applying the metabolomic approach in clinical research.

## 1. Introduction

Osteoarthritis (OA) is one of the most common causes of lameness in sport horses [1]. This condition, especially when persistently affecting the fetlock joint, can impair horse athletic activity and welfare, resulting in significant economic consequences [2].

The pathogenesis of osteoarthritis involves both mechanical factors and chemical mediators, the latter represented by interleukins, enzymes, free radicals, and antibodies [1]. Both mechanical and chemical insults lead to the breakdown and loss of proteoglycans, that, in turn, alter the physical property of cartilage, making it more susceptible to further damage [1].

During OA, different pathobiological processes occur at the same time. Physical and/or biochemical damage of the articular cartilage and bone initiates the inflammation of the synovial membrane, articular bone surfaces, and fibrous joint capsule (synovitis and capsulitis). The synovial membrane is usually involved early in the process, but it remains unclear whether its modifications occur first or whether they are the result of cartilage degradation or lesions of the subchondral bone. Several cell types have been pointed out to be responsible for synovial inflammation, but studies have shown that much of the inflammation in the OA joint is attributed to synovial proinflammatory macrophages [3]. Catabolic and proinflammatory mediators, such as cytokines (interleukin-1β, interleukin-6, and tumor necrosis factor α), nitric oxide, prostaglandin E2, and neuropeptides, produced by the inflamed synovium, may further stimulate cartilage extracellular matrix (ECM) degradation, which creates a hurtful circle [4]. Cartilage ECM degradation can also be the result of direct physical damage to the articular cartilage and subchondral bone, and an imbalance between the synthesis and breakdown of such tissues may lead to the secretion of proinflammatory cytokines, which cause, in turn, cartilage degradation [5]. In addition, exposure to inflammatory and oxidative mediators enhances premature stress-induced senescence and the aging of chondrocytes [6].

At present, no effective treatment has been identified, so therapy is aimed merely at managing lameness itself, with palliative systemic anti-inflammatory drugs and local injections of substances such as corticosteroids, hyaluronan, or interleukin receptor antagonist protein (IRAP) [1].

A step forward in the prevention of osteoarthritis may come from its early diagnosis and from the possibility of monitoring its evolution to prevent the worsening of the horse’s clinical condition and preserve athletic activity [2]. The scientific community has been working to discover biomarkers and differentiate septic from nonseptic arthritis, thus identifying the underlying causes with the aim of better understanding the pathophysiology of osteoarthritis in horses [2,7,8,9].

Synovial fluid (SF) acts as a lubricant of the joints and therefore is at direct contact with the sore spots. For these reasons, in the diagnostic workup in addition to clinical and radiographic assessments, total proteins and white blood cells are routinely investigated by practitioners for the diagnosis of joint diseases [10,11].

In recent years, omics sciences, from genomics downwards to metabolomics, have granted a wealth of new diagnostic approaches in clinical and biomedical studies thanks to continuous developments in analytical techniques and bioinformatics [12,13], these approaches have also impacted the study of joint diseases through the observation of the synovial fluid in equines as well as in humans [14,15,16,17,18].

Among the omics platforms, metabolomics seems perfectly suited to biomarker identification, being the one dedicated to the characterization of low-weight metabolites, the so-called metabolome, the most directly altered by inflammatory processes [19]. Metabolomics investigations have targeted both the lipidic and the water-soluble metabolome’s fractions. An example of the study of the lipidic fraction can be traced in the work of De Grauw et al. [20], who applied it to study the release of eicosanoid acid in synovial joints, or Kosinska and colleagues [21], who performed a comparative study on the lipidome of normal knee synovial fluid from humans and horses. Water-soluble metabolome was investigated by Anderson et al. [2] who studied the fingerprints on the SF’s metabolome of septic and nonseptic joint pathologies. The authors applied ^1^H-NMR spectroscopy in an untargeted manner and, promisingly, they identified as many as 55 metabolites, mainly pertaining to the chemical groups of amino acids and derivatives and to organic acids. The SF’s metabolome resulted in being extensively affected by sepsis, with 26 metabolites differently concentrated compared to nonseptic counterparts. In 2020, the same group confirmed the richness of information granted by metabolomics based on ^1^H-NMR by successfully studying palmar osteochondral disease [22].

These premises allow us to foresee the richness of information granted by ^1^H-NMR on the water-soluble metabolome of SF also in the case of osteoarthritis. Unfortunately, the applications of this technique to the purpose are restricted to the work of Lacitignola et al. [23], who limited themselves to the targeted quantification 10 molecules, probably due to limitations in the hardware used and databases it was possible to consult when the work was published.

Considering the importance of obtaining an in-depth knowledge of equine osteoarthritis pathophysiology, we have applied ^1^H-NMR in an untargeted manner to investigate as comprehensively as possible the differences in synovial fluid composition between healthy and OA-affected joints in horses.

## 2. Materials and Methods

### 2.1. Animals

Synovial fluid was collected from the metacarpophalangeal joints (fetlock) of 19 horses of different breeda, 11 animals affected by osteoarthritis (OA group), and 8 healthy horses slaughtered for meat production.

Osteoarthritis was diagnosed in the OA Group (6 males and 5 females of 7.6 ± 2.1 years) by a combination of clinical examination, radiographic and ultrasonographic findings, SF total protein, and cytology, as previously described [1,24].

The day before slaughtering, the horses from Group C (5 males and 3 females of 3.9 ± 0.8 years) were subjected to clinical and radiographic examinations to ascertain that animals were free of lameness and radiographic abnormalities.

Briefly, after the proper preparation of the fetlock region, SF samples were obtained aseptically by arthrocentesis using a 21-gauge needle and a 2.5 mL syringe. Fetlock arthrocentesis was performed with the limb in flexion and with the needle inserted into the area between the third metacarpal bone and the suspensory ligament of the fetlock. The needle was introduced into the joint to a depth of about 1 cm [1].

The collected samples were then transferred to 1.5 mL tubes and processed within one hour of the aspiration procedure. Subsequently, the tubes were centrifuged (at 4 °C, 2540× *g* for 5 min), the cellular component was eliminated, and, subsequently, the supernatant was transferred into another 1.5 mL tube. The samples were then frozen and stored at a temperature of −80 °C until analysis.

SF samples from group C were taken immediately after slaughtering (within 10 min), with the same described procedure occurred for group OA.

All experimental procedures were approved by the Institutional Animal Care and Use Committee of Camerino University (Registration number: 1/2023) and were in accordance with the standards recommended by the EU Directive 2010/63/EU for experiments on animals.

### 2.2. Metabolome Observation by ^1^H-NMR

To investigate the metabolome of synovial fluids by ^1^H-NMR, a solution based on D_2_O was first created, set at pH 7.00 ± 0.02 by means of a phosphate buffer. It contained 3-(trimethylsilyl)-propionic-2,2,3,3-d4 acid sodium salt (TSP) 10 mmol/L, as an NMR chemical-shift reference, and NaN_3_ 2 mmol/L, that avoided bacterial proliferation.

Following Brugaletta et al. [25], each sample of synovial fluid was thawed and then centrifuged at 18,630× *g* for 10 min at 4 °C. An aliquot of 0.35 mL of supernatant was added to 0.35 mL of deionized water and to 0.1 mL of the above-described NMR solution. After a further centrifugation at the above conditions, a spectrometer AVANCE III (Bruker, Milan, Italy), set at a frequency of 600.13 MHz and operated by the software Topspin (v3.5), was used to register the spectra at 298 K. Broad resonances’ signals due to large molecules were suppressed by a CPMG-filter, composed of 400 echoes spaced by 0.400 ms, and obtained with 180 pulses of 24 µs for a total filter of 330 ms. The residual signal from water was suppressed by presaturation. This was obtained by relying on the cpmgpr1d sequence, part of the vendor’s standard library of experiments. Each spectrum was the sum of 256 transients, sampled with 32,000 data points over a 7184 Hz spectral window, with an acquisition time of 2.28 s and a recycle delay of 5 s.

The ^1^H-NMR spectra were adjusted for phase and baseline in Topspin and then exported to ASCII format through the corresponding Bruker’s script. The subsequent steps were performed in R [26] through custom scripts. The assignment of signals was performed by comparing their position in the spectra and shape and multiplicity with the Human Metabolome Database [27] and Chenomx software libraries (Chenomx Inc., Edmonton, AB, Canada, v10) via the routines made available by Chenomx software. The absolute concentration of molecules was obtained in the sample with the median water dilution, assessed through probabilistic quotient normalization [27], by relying on the TSP area and known concentration. Differences in water content across the set of samples were considered through probabilistic quotient normalization. The concentration of each molecule was obtained from the area of one of its signals, calculated by rectangular integration.

### 2.3. Statistical Analysis

To ensure the normal distribution of the data, Box–Cox transformation was used [28]. A *t*-test was subsequently applied to evaluate statistical differences in the concentrations of the various metabolites detected in the synovial fluid in the two groups of horses. Statistical analysis was not corrected for multiple testing. A *p* value ≤ 0.05 was considered statistically significant. Furthermore, the rPCA model and volcano plot were performed on the molecules found to be significantly different between AO and control samples.

## 3. Results

**Figure 1:**
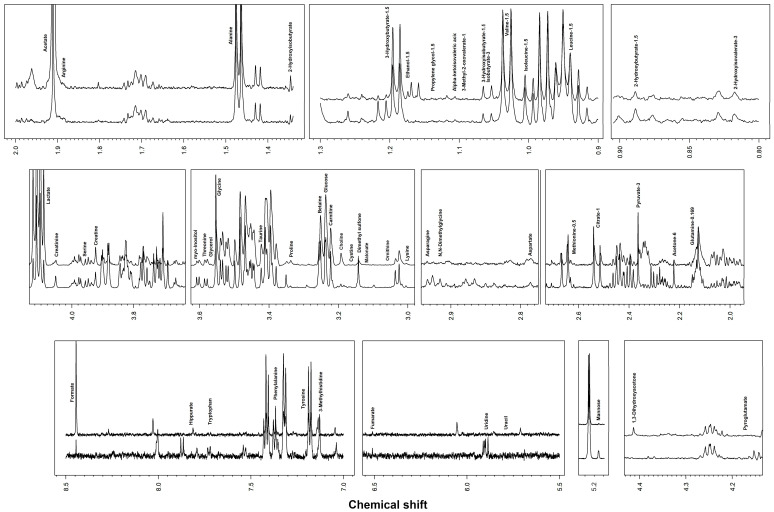
Representative spectra of equine synovial fluid.

Metabolomic analysis by ^1^H-NMR allowed 55 different metabolites to be identified and quantified in SF samples (Figure 1).

Nineteen metabolites were found to be differently concentrated in the OA group compared to the control group. Each metabolite’s concentration is shown in Table 1. The synovial fluids from OC group, compared to those of the control group, were found to be richer in 1,3-dihydroxyacetone but poorer in tryptophan, phenylalanine, tyrosine, uridine, creatinine, creatine, glycine, choline, asparagine, glutamine, arginine, 3-hydroxybutyrate, valine, 2-hydroxyisovalerate, α-ketoisovaleric acid, 3-methyl-2-oxovalerate, and methionine.

**Figure 2:**
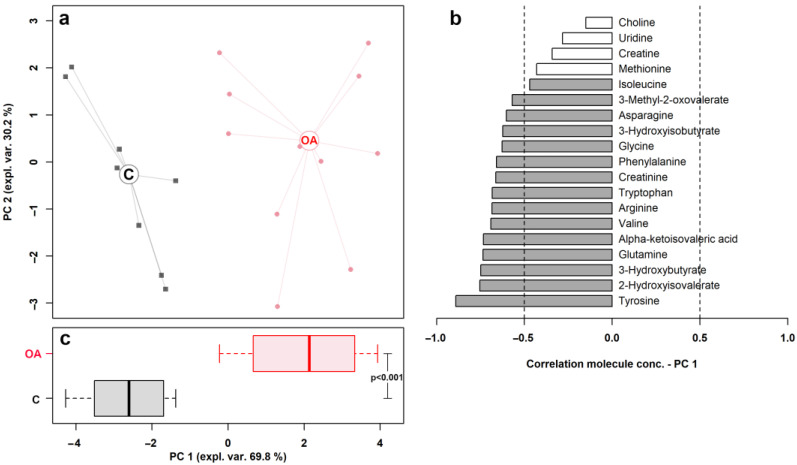
rPCA model built on the space constituted by the concentration of the molecules showing a significant difference between control (C) and osteoarthritis (OA) groups. In the scoreplot (**a**), samples from C and OA groups are represented with squares and circles, respectively. The wide, empty circles represent the median of the group. The loading plot (**b**) reported the correlation between the concentration of each substance and its importance over PC 1; significant correlations (*p* < 0.05) are highlighted with gray bars. The bar plot (**c**) summarized the positions of the samples along PC 1.

**Figure 3:**
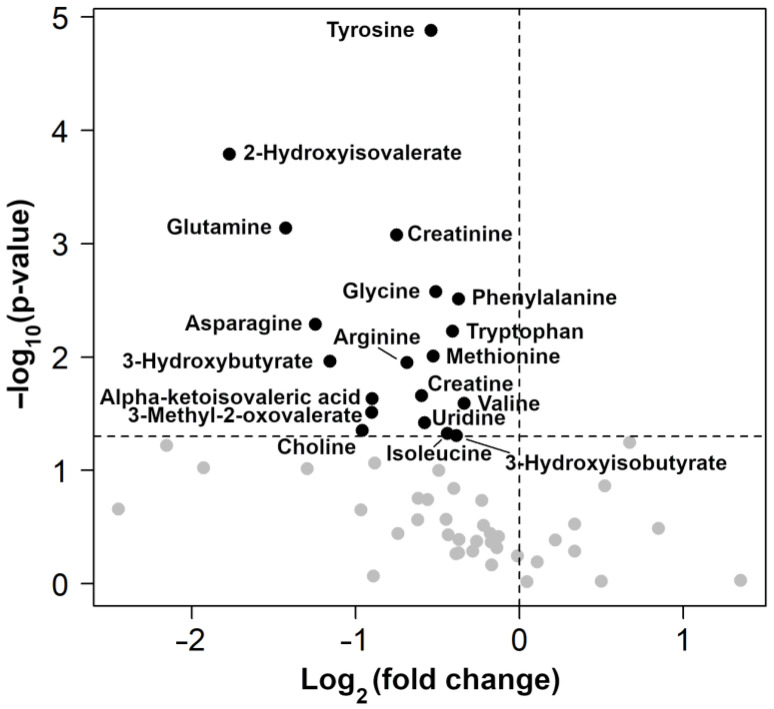
Volcano plots showing the change between control and osteoarthritis (OA) groups in all metabolites identified. Black dots with names are used only for molecules with significantly different concentrations between groups.

The results of the rPCA model and volcano plot are summarized in Figure 2 and Figure 3, respectively.

## 4. Discussion

This study highlighted the differences in the metabolomic profile of SF from healthy joints compared to joints affected by OA. By relying on the untargeted application of ^1^H-NMR as an analytical platform, as many as 55 metabolites were identified and quantified, 21 of which showed statistically different concentrations between the OA and control group. Significant variations involved tryptophan, phenylalanine, tyrosine, glycine, asparagine, glutamine, arginine, methionine, and 1,3-Dihydroxyacetone.

In our study, tryptophan was less concentrated in the OA group compared to the control group. This metabolite was found to exert antiproliferative properties along the kynurenic acid pathway against rabbit and human synovial fibroblasts [29], thus suggesting that it plays a role in OA pathogenesis. According to the current literature, the concentration of tryptophan and its catabolites changes according to inflammatory processes [30]. Tryptophan and its catabolites, namely kynurenic and quinaldic acid, were found in the SF of human patients affected by joint diseases and resulted to be lower in patients with rheumatoid arthritis (RA) than those with OA [29]. Increased kynurenic acid levels were also observed in the SF of patients with OA and in those affected by RA [31]. In a recent study on horses with carpal OA, higher concentrations of tryptophan were found to be associated with a decrease in kynurenine [18].

Phenylalanine and tyrosine also showed a marked decrease in the OA group in our study. Phenylalanine, an essential amino acid, can be converted into tyrosine that, in turn, can be converted into L-DOPA, thyroxine, adrenaline, and norepinephrine. It is also involved in the regulation of enzyme activities. A study by Li and colleagues [32] analyzed the SF of human patients with RA, finding that L-phenylalanine had higher concentrations in the SF of healthy subjects compared to RA patients [32]. These results are in agreement with the phenylalanine levels recorded in our study, which were found to be higher in the SF samples of healthy horses. Anderson et al. [2] performed a metabolomic analysis of SF collected from different joints affected by OA (*n* = 4), osteochondrosis (*n* = 6), and synovial sepsis (*n* = 7) in horses. The authors found higher concentrations of phenylalanine in the SF from nonseptic compared to septic joints, but no comparison was available with healthy joints for the lack of a control group. In this context, the role of phenylalanine in joint diseases and the meaning of its variation in horses needs to be clarified.

Glycine was lower (*p* < 0.01) in the OA group compared to the controls. This nonessential amino acid is characterized by numerous functions, including the synthesis of proteins, in particular collagen and elastin, and plays a fundamental role in the synthesis of ATP. Collagen is the main protein of connective tissue, and its structural unit is tropocollagen, a protein formed by three polypeptide chains. All tropocollagen molecules are constituted by the same sequence of amino acids: glycine, proline, and a third amino acid or glycine, hydroxyproline, and a third amino acid. Since collagen (especially type II collagen) is the main protein constituting cartilage, its presence is a fundamental factor for the preservation of joints. In a study conducted in 2018 by Dai et al. [33], it was observed that the increase in collagen and glycine prevented the onset of joint pathologies in rats, with specific regard to osteoarthritis. Low concentrations of this amino acid in the SF of pathological joints lead to a decrease in collagen and cartilage and consequently joint damage.

Asparagine is a nonessential polar amino acid, which in our study decreased in the OA group. Despite the fact that knowledge on its effect on joints is lacking, it is known to be involved in the urea cycle, in gluconeogenesis, and in the synthesis of neurotransmitters. There is some evidence that the removal of asparagine from culture medium (from lymphoma and colon tumor) impairs cell proliferation and induces cell cycle arrest [34]. On this basis, it can be speculated that cell proliferation in joint disease could be affected by SF asparagine, although its role in joint metabolism is not well understood and needs further investigations.

Another nonessential amino acid, glutamine, decreased in the SF of the OA group. Biologically, this metabolite is involved in various biological functions, including the transporter of amino groups, precursor of glutamate and glutathione, energy support for lymphocytes, macrophages and enterocytes, stimulator of nucleotide synthesis, and the inducer of the expression of heat shock proteins (HSPs), capable of decreasing the inflammatory response by cytokines. Thermal shock proteins have a fundamental protective function, and a lack of these proteins can lead to cellular apoptosis and more extensive tissue damage [35]. In 2017, a study conducted by Takahashi et al. demonstrated a higher proliferation of fibroblast-like synoviocytes following the exogenous administration of glutamine in rats and a greater consumption of glutamine in subjects with inflammatory joint diseases [36]. These findings agree with our results showing lower concentrations of this amino acid found in horses from the OA group.

In our study, we found arginine, an amino acid classified as conditionally essential, less concentrated in the SF from the OA group. This metabolite has beneficial effects as it promotes the oxidation of fatty acids, increases the activity of lipolytic enzymes, and reduces insulin resistance [37]. Arginine is also a substrate in the NO cycle and its metabolism has been described as downregulated during OA, providing further support of altered NO metabolism. In addition, arginine, together with citrulline, is also an intermediary molecule in the urea cycle, which produces NO as a byproduct via nitric oxide synthase. These results suggest that oxidative stress plays a role in the pathogenesis of OA in horses as well as that of RA in humans [38].

Methionine is an essential nonpolar amino acid with a strong antioxidant and protein structure stabilizing action [39]. A recent study by Li et al. [40] investigated the role of some amino acids, including methionine, in human joint disease, asserting that a decrease in the concentration of amino acids is mainly related to increased consumption during joint inflammatory processes [40]. Our study confirmed lower levels of methionine in the SF of horses with OA.

Creatine and creatinine were found to be lower in the SF from the OA group compared to healthy subjects. Another study by Anderson and colleagues [2] found higher concentrations in SF during nonseptic joint disease than in septic process. However, the role of these metabolites, mainly known to be involved in energetic metabolism, should be further investigated during articular diseases.

Finally, 1,3-Dihydroxyacetone was the only metabolite with a higher concentration in the SF of the OA group in comparison to the control group. To explain this, different theories can be adopted. First, this finding can be related to an alteration of energetic metabolism during the OA process as dihydroxyacetone phosphate (DHAP), the phosphorylated form, takes part in glycolysis and is an intermediate product of glucose metabolism. In addition to this, 1,3-Dihydroxyacetone has been demonstrated to be capable of enhancing GAG accumulation in cultured human articular chondrocytes and cartilage tissue, and it can be speculated that the increase in such metabolites represents an attempt from chondrocytes to attenuate the detrimental effect of OA. In 2001, Liu and collaborators [41] demonstrated that hexosaminidase is the dominant glycosaminoglycan (GAG)-degrading glycosidase released by chondrocytes into the extracellular compartment, and it is the dominant glycosidase in the synovial fluid of patients with osteoarthritis. During OA, different cytokines are secreted, and it has been fully elucidated that the stimulation of chondrocytes with pro-inflammatory cytokines, especially interleukin-1β, results in a selective secretion of hexosaminidase that causes both a modification of glucose metabolism and GAG degradation [42,43]. To test the hypothesis that hexosaminidase serves as one of the key enzymes participating in cartilage matrix GAG degradation, Liu and collaborators synthesized the iminocyclitol inhibitors of hexosaminidase, starting from the aldol addition reaction of an aldehyde with dihydroxyacetone phosphate catalyzed by fructose-1,6-diphosphate aldolase. As a result, the inhibition of hexosaminidase resulted in a decrease in GAG degradation, and this fact should be taken into account when planning future strategies to mitigate and prevent OA-related occurrences.

The alteration in joint metabolism during OA can also explain the differences observed in other SF metabolites like 2-hydroxyisovalerate, 3-hydroxybutyrate, 3-hydroxyisobutyrate, α-ketoisovaleric acid, and 3-methyl-2-oxovalerate, choline, uridine, and valine.

2-hydroxyisovalerate, 3-hydroxybutyrate, 3-hydroxyisobutyrate, and α-ketoisovaleric acid are organic hydroxy acids representing the intermediate metabolites of amino acid metabolism, found to be overexpressed during OA in human joints [44].

On the human side, a number of studies have clarified that OA is responsible for an activation of specific amino acid metabolic pathways, causing an increase in the SF levels of organic acid as 2-hydroxyisovalerate, 3-hydroxybutyrate, and 3-hydroxyisobutyrate [44].

In our study, we found a contrary trend of such metabolites during OA in horses.

Adams and colleagues [45] recently applied global metabolic profiling to identify the metabolic profile of cultured human synovial tissue from patients with end-stage OA compared to patients with little or no evidence of disease. A key difference between SF from normal and OA joints was related to the marked different branched-chain amino acid (BCAA) catabolism. In general, they found that the cultured synovial tissues acted to increase the amino acid contribution to the media. By contrast, the levels observed for tryptophan and the BCAAs, as leucine, isoleucine, and valine, in conditioned media samples, did not differ from the control media, while catabolic products of the BCAAs were evident in both late disease and early/no disease groups. 3-methyl-2-oxovalerate is a first-step catabolite of BCAAs revealed to be present in all samples from tissue culture, indicating that its occurrence is largely via cellular metabolism and excretion.

However, a lower level of BCAAs and organic acid metabolites in media from the late-stage OA group may be explained by an alteration in BCAA metabolism with disease progression. Our findings support this hypothesis. Recently, the ratio of BCAA to the amino acid histidine was proposed as a biomarker for OA [46]. In that study, increased serum BCAA was correlated with the radiographic severity of OA, suggesting altered BCAA metabolism plays a role in OA.

From a biochemical point of view, choline and uridine are methyl donors that can modulate methylation in different metabolisms. As an example, via betaine homocysteine methyltransferase, choline regulates the concentrations of S-adenosylhomocysteine and S-adenosylmethionine which have been shown to help the regeneration of cartilage tissue during OA, and dietary supplementation with SAMe represents a main strategy for cartilage restoration [47].

Recently uridine has received attention for its anti-inflammatory properties and its potential as an antiarthritic therapy in an experimental model of rheumatoid arthritis (RA) [43]. In this model, arthritis was induced by the intra-articular injection of the antigen in pre-sensitized mice, which results in inflammation resembling RA in terms of synovial membrane hyperplasia, leukocyte infiltration, and pannus formation. Then, Narendra and collaborators locally administered uridine which was able to trigger a dramatic suppression of synovial ICAM-1, CD18 expression, and the local expression of pro-inflammatory cytokines in a dose-dependent manner.

Finally, lower concentrations of choline have been found in the OA group compared to the controls. This metabolite is involved in the cholinergic anti-inflammatory pathway that seemed to improve the progression of OA disease [48].

## 5. Conclusions

The present study investigated the metabolomic profile of SF in horses affected by osteoarthritis and in healthy horses by demonstrating the variety of molecules that undergo significant changes during joint disease. We can speculate that the different concentration of certain metabolites could result from a reduced efficiency of homeostatic control mechanisms balancing pro- and anti-inflammatory pathways in OA joints. The possibility of identifying potential biomarkers and exploring the mechanism underlying osteoarthritis in horses represent the main reasons for promoting the application of the metabolomic approach in clinical research, and every metabolomic study will contribute towards reaching this goal.

## Figures and Tables

**Table 1:** Metabolites found in synovial fluid samples from horses included in the study. Values are expressed as mean ± standard deviation (SD). Statistical significances between control and osteoarthritis (OA) groups are in bold.

Metabolite	Concentration (mmol/L)		*p* Value
	Control Group	OA Group	
1,3-Dihydroxyacetone	0.003 ± 0.001	0.006 ± 0.004	**0.03**
2-Hydroxybutyrate	0.01 ± 0.007	0.007 ± 0.004	0.18
2-Hydroxyisobutyrate	0.033 ± 0.008	0.036 ± 0.008	**0.03**
2-Hydroxyisovalerate	0.007 ± 0.002	0.003 ± 0.002	**0.0001**
3-Hydroxybutyrate	0.317 ± 0.115	0.175 ± 0.121	**0.01**
3-Hydroxyisobutyrate	0.017 ± 0.01	0.012 ± 0.012	0.05
3-Methyl-2-oxovalerate	0.004 ± 0.002	0.002 ± 0.001	**0.02**
3-Methylhistidine	0.154 ± 0.02	0.147 ± 0.05	0.34
Acetate	0.966 ± 0.731	0.721 ± 0.542	0.22
Acetone	0.014 ± 0.007	0.162 ± 0.49	0.17
Alanine	0.629 ± 0.043	0.59 ± 0.136	0.19
Arginine	0.226 ± 0.027	0.155 ± 0.075	**0.006**
Asparagine	0.079 ± 0.03	0.039 ± 0.024	**0.004**
Aspartate	0.051 ± 0.013	0.089 ± 0.07	0.06
Betaine	0.096 ± 0.033	0.071 ± 0.020	0.06
Carnitine	0.033 ± 0.009	0.032 ± 0.022	0.43
Choline	0.041 ± 0.019	0.025 ± 0.017	**0.03**
Citrate	0.602 ± 0.136	0.526 ± 0.251	0.12
Creatine	0.201 ± 0.038	0.145 ± 0.063	**0.01**
Creatinine	0.161 ± 0.025	0.101 ± 0.039	**0.0004**
Cystine	0.011 ± 0.008	0.021 ± 0.057	0.29
Dimethyl sulfone	0.069 ± 0.028	0.105 ± 0.126	0.19
Ethanol	0.0343 ± 0.872	0.025 ± 0.042	0.17
Formate	0.043 ± 0.006	0.072 ± 0.085	0.14
Fumarate	0.009 ± 0.001	0.009 ± 0.002	0.45
Glucose	7.296 ± 1.453	11.317 ± 7.441	0.06
Glutamine	0.450 ± 0.102	0.205 ± 0.169	**0.001**
Glycerol	0.047 ± 0.022	0.039 ± 0.028	0.25
Glycine	1.246 ± 0.088	0.906 ± 0.270	**0.001**
Isobutyrate	0.002 ± 0.002	0.001 ± 0.001	0.08
Hippurate	0.04 ± 0.018	0.028 ± 0.011	0.07
Isoleucine	0.128 ± 0.022	0.101 ± 0.047	0.05
Lactate	8.076 ± 3.231	5.733 ± 5.532	0.13
Leucina	0.322 ± 0.05	0.319 ± 0.164	0.48
Lysine	0.131 ± 0.014	0.128 ± 0.08	0.45
Malonate	0.003 ± 0.002	0.003 ± 0.003	0.47
Mannose	0.108 ± 0.033	0.093 ± 0.063	0.25
Methionine	0.048 ± 0.005	0.035 ± 0.015	**0.01**
Myo-inositol	0.069 ± 0.065	0.044 ± 0.027	0.17
N,N-Dimethylglycine	0.004 ± 0.001	0.003 ± 0.002	0.27
Ornithine	0.008 ± 0.005	0.004 ± 0.005	0.06
Phenylalanine	0.555 ± 0.047	0.437 ± 0.094	**0.001**
Proline	0.198 ± 0.058	0.276 ± 0.138	0.06
Propylene glycol	0.011 ± 0.004	0.061 ± 0.107	0.08
Pyroglutamate	0.028 ± 0.013	0.023 ± 0.013	0.24
Pyruvate	0.089 ± 0.04	0.069 ± 0.047	0.17
Serine	0.323 ± 0.039	0.296 ± 0.084	0.19
Taurine	0.006 ± 0.005	0.005 ± 0.037	0.31
Threonine	0.36 ± 0.08	0.306 ± 0.062	0.07
Tryptophan	0.059 ± 0.007	0.046 ± 0.012	**0.003**
Tyrosine	0.179 ± 0.013	0.125 ± 0.024	**0.00001**
Uracil	0.025 ± 0.003	0.027 ± 0.008	0.15
Uridine	0.045 ± 0.021	0.028 ± 0.008	**0.03**
Valine	0.437 ± 0.059	0.355 ± 0.087	**0.01**
α-ketoisovaleric acid	0.007 ± 0.003	0.004 ± 0.002	**0.02**

## Data Availability

Data available by the corresponding author upon reasonable request, Data are not publicly available due to privacy.
